# Understanding short-timescale neuronal firing sequences via bias matrices

**DOI:** 10.1186/1471-2202-16-S1-P108

**Published:** 2015-12-18

**Authors:** Zachary J Roth, Yingxue Wang, Eva Pastalkova, Vladimir Itskov

**Affiliations:** 1 Department of Mathematics, University of Nebraska-Lincoln, Lincoln, NE 68588, USA; 2 Janelia Research Campus, HHMI, Ashburn, VA 20147, USA; 3 Department of Mathematics, The Pennsylvania State University, University Park, PA 16802, USA

## 

The brain generates persistent neuronal firing sequences across varying timescales. The short-timescale (~100ms) sequences are believed to be crucial in the formation and transfer of memories. Large-amplitude local field potentials known as sharp-wave ripples (SWRs) occur irregularly in hippocampus when an animal has minimal interaction with its environment, such as during resting, immobility, or slow-wave sleep. SWRs have been long hypothesized to play a critical role in transferring memories from the hippocampus to the neocortex [1]. While sequential firing during SWRs is known to be biased by the previous experiences of the animal, the exact relationship of the short-timescale sequences during SWRs and longer-timescale sequences during spatial and non-spatial behaviors is still poorly understood. One hypothesis is that the sequences during SWRs are "replays" or "preplays" of "master sequences", which are sequences that closely mimic the order of place fields on a linear track [2,3]. Rather than particular hard-coded "master" sequences, an alternative explanation of the observed correlations is that similar sequences arise naturally from the intrinsic biases of firing between pairs of cells. To distinguish these and other possibilities, one needs mathematical tools beyond the center-of-mass sequences and Spearman's rank-correlation coefficient that are currently used.

**Figure 1:**
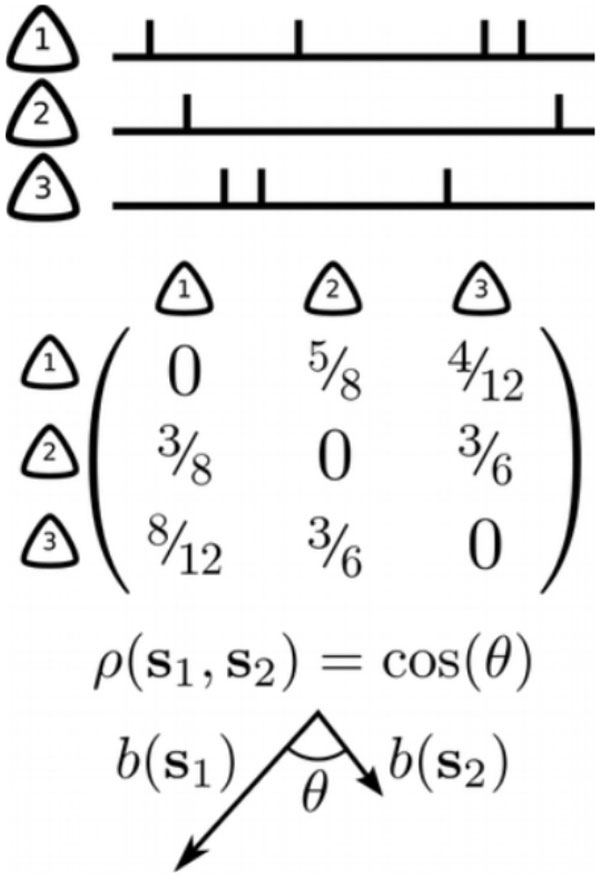
**Neuronal spike trains (top) are converted to bias matrices (middle) by computing the probability of pairs of neurons spiking in a particular order**. The correlation between bias matrices (bottom) is then computed via the angle between the bias matrices.

We introduce a new mathematical tool that captures the intrinsic properties of neuronal firing sequences. The *bias matrix *of a given sequence (Figure 1) contains more detailed information than the center-of-mass average and captures more complex relationships among different neuronal sequences. This tool enabled us to directly investigate the relationships among firing sequences across different conditions: short-timescale sequences (during SWRs) and long-timescale behavioral sequences (during spatial navigation and wheel running). We also performed a pharmacological manipulation that resulted in elimination of theta oscillation (as previously reported in [4]) and increased the frequency of SWRs. We have found that the pairwise biases of sequences during SWRs are highly correlated with sequences during most of the conditions. Moreover, while sequences of neuronal activations are uncorrelated across different behaviors, the bias matrices of SWR sequences are highly correlated with those of various behavior sequences. Our findings provide a new tool for understanding the structure of short-timescale neuronal sequences and suggest that intrinsic pairwise biases are likely the underlying mechanism for the "replay/preplay" of longer-timescale sequences observed in the hippocampus [2,3].
